# Percutaneous Cava Stenting in a Dog with Symptomatic Azygos Continuation of the Caudal Vena Cava

**DOI:** 10.1155/2020/7523247

**Published:** 2020-08-17

**Authors:** Giovanna Bertolini, Marco Caldin

**Affiliations:** ^1^Diagnostic and Interventional Radiology Division, San Marco Veterinary Clinic and Laboratory, Via dell'Industria, 35030 Veggiano (Padova), Italy; ^2^Clinical Pathology Division, San Marco Veterinary Clinic and Laboratory, Via dell'Industria, 35030 Veggiano (Padova), Italy

## Abstract

This report describes the successful placement of a nitinol stent within the azygos continuation of the caudal vena cava in a 2-year-old, neutered female, English Bulldog with clinical and imaging signs related to venous return chronic obstruction, renal venous thrombi, and chronic renal insufficiency. This noninvasive, interventional radiology procedure was safe and clinically effective for the patient. The clinical signs were rapidly eliminated, and three years later, the patient is still in good clinical condition, with normal renal function. Venous stenting appears to be a useful, new, minimally invasive treatment option for symptomatic cavo-azygos vascular connection.

## 1. Introduction

In the developing embryo, the caudal vena cava (CVC) and azygos vein are result of selective anastomoses, persistence, and degeneration of three vascular precursors [1]: the supracardinal, subcardinal, and vitelline veins. In normal mammals, the CVC is divided into five segments: prerenal, renal, prehepatic, hepatic, and posthepatic. According to the supracardinal theory, the prerenal segment of the CVC develops from the right subcardinal vein and the hepatic segment of the CVC is derived from the vitelline veins [2]. In the thoracic region, the supracardinal veins give rise to the azygos and hemiazygos veins. Thus, both the prerenal segment of the CVC and the azygos vein system are derived from the supracardinal vein. The failure to form the right subcardinal-vitelline anastomosis in the developing caudal vena cava in the embryo results in segmental “interruption of the CVC with azygos continuation.” As a consequence, blood is redirected from the supra-subcardinal anastomosis, at the renal level, through the thoracic azygos vein system [1, 2].

Once considered rare, azygos-cava shunts are now often encountered in practice, because of the increasingly widespread use of advanced imaging techniques in small animals, although the prevalence is not known [3–5]. The azygos continuation of the CVC is usually considered an incidental finding without clinical consequences. However, complex embryological defects may occur in the same patient and accompany the cavo-azygos communication, such as portosystemic shunts with or without concurrent portal vein aplasia [4–6], or maybe complicated by thrombus formation [3, 4].

## 2. Case Presentation

A 2-year-old, neutered female, English Bulldog weighing 17.8 kg presented to the referring veterinarian with a chronic renal insufficiency diagnosed in the previous year revealed a possible vascular anomaly on abdominal ultrasound. The dog was then referred to the authors' tertiary hospital for further characterization of the vascular anomaly with computed tomography. A multiphase vascular examination was obtained using a third-generation dual-source computed tomography (192 × 2 DSCT) (Somatom Definition Force; Siemens, Erlangen, Germany). CT settings were as follows: one tube for arterial and interstitial phase (120 kVp) and two tubes for portal venous phase at 100/150 kVp. For all phases, 400 mAs/rot (0.28 sec), collimation 192 × 0.6 mm; images were reconstructed at 0.3 mm using a soft-tissue reconstruction algorithm.

On physical examination, the dog showed no abnormal physiologic findings. Results of the hematobiochemical and urinary examinations showed a moderate inflammatory status (c-reactive protein 0.58 mg/dl (0.01-0.10 mg/dl)) associated to hyperazotemia (urea 70 mg/dl (19-53 mg/dl) and creatinine 2.08 mg/dl (0.75-1.33 mg/dl)) and decreased urine concentration (osmolality 490 mOsm/kg (925-1934 mOsm/kg)). Again, the dog showed a marked proteinuria (PU/CU 3.2 (0.1-0.2)). All this information was consistent with the known chronic renal failure.

**Figure 1:**
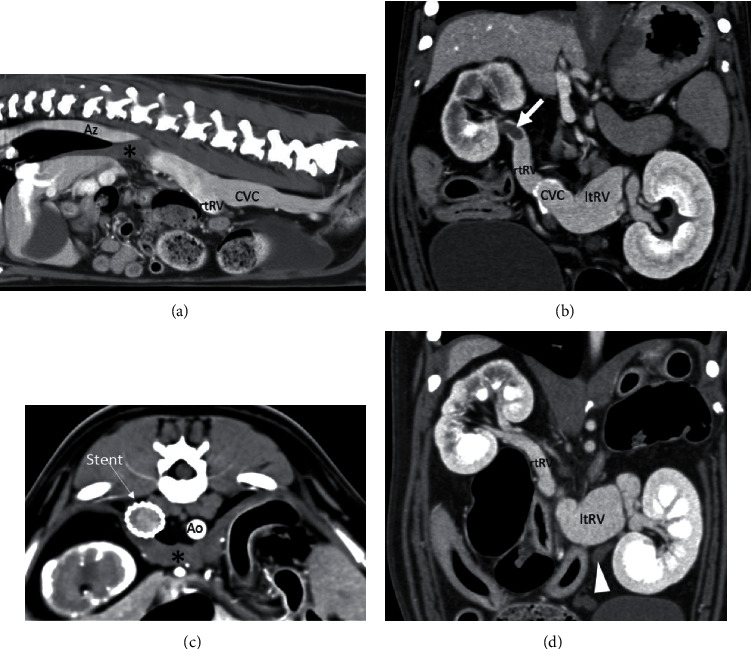
Multiphase CT examination on admission and after stent placement. (a) Sagittal multiplanar reformatted (MPR) image showing the absence of the prerenal segment of the caudal vena cava (CVC) with azygos continuation (Az). The asterisk indicates the diaphragmatic crura; rtRV: right renal vein. (b) Dorsal MPR image. Note the thrombus in the right renal vein (arrow) and engorged left renal vein (ltRV). (c) Transverse view after stent placement. Ao: descending aorta. (d) Dorsal MPR after stent placement. Note the normally perfused right renal vein. The left one is still enlarged. The arrowhead indicates the enlarged left gonadal vein (not enhanced).

**Figure 2:**
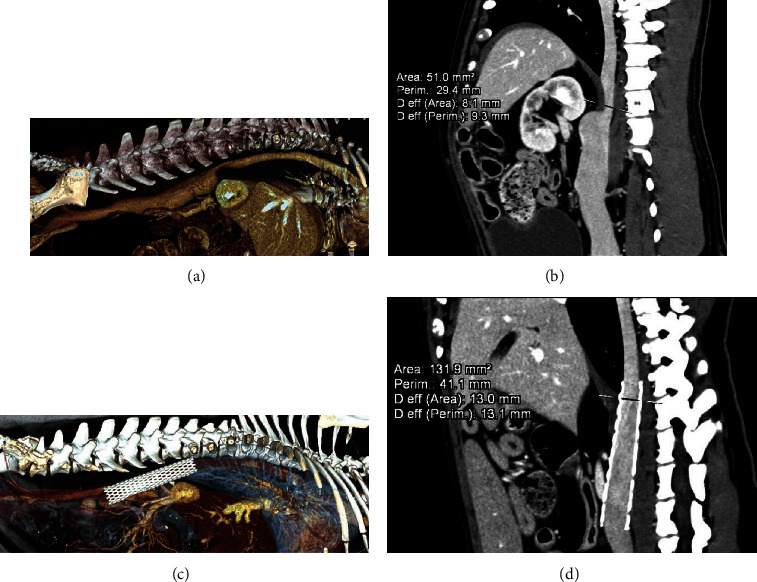
(a) Volume rendering image from CT data from right point of view. Note the stenosis of the CVC at the thoracoabdominal passage. (b) Quantification of the vessel characteristics at this level. (c) VR image, right point of view, after stent placement. (d) Note the uniform, reduced CVC diameter.

The multiphase computed tomography of the body revealed dilatation of both the iliac veins. The prerenal and renal segments of the caudal vena cava were engorged. Both the renal veins were dilated, and there was aneurysmal dilatation at the joining level of the left renal vein and the renal segment of the cava. Again, there was a 15 mm filling defect in the right renal vein and multiple, small filling defects in the left renal vein lumen (Figures 1(a) and 1(b)). Both kidneys had altered shape and appearance and showed a transitory nonhomogenous enhancement in the corticomedullary phase, normalized in the excretory phase. The prehepatic segment of the caudal vena cava was absent, and its renal segment communicated dorsally with the right azygos vein. This latter was distended and received a dilated left hemiazygos vein at the level of the 10^th^ thoracic vertebra. At the diaphragmatic passage, the anomalous vascular channel showed more than 50% of reduction in diameter (Figures 2(c) and 2(d)). The hepatic and posthepatic caudal vena cava was normally represented. Again, the absence of the external left jugular vein was noted. Multiple venous-venous collaterals involving the gonadal, epigastric-pudendal, and intercostal veins were also detected.

Based on imaging findings, the azygos continuation of the caudal vena cava (due to segmental aplasia of the prehepatic segment) was diagnosed, with stenosis at the diaphragmatic passage, associated to direct and indirect signs of chronic caudal vena cava obstruction and its complications: aneurysmal dilatation of the cava (prerenal and renal) and renal veins, renal venous thrombosis, and cava collateral formation. A venous stent placement in the stenotic passage under fluoroscopic guidance was then planned on day four.

For peri-interventional venous thromboembolism prevention, heparin was subcutaneously administered the day of the interventional procedure and the five days after (dalteparin 100 UI/kg SID).

**Figure 3:**
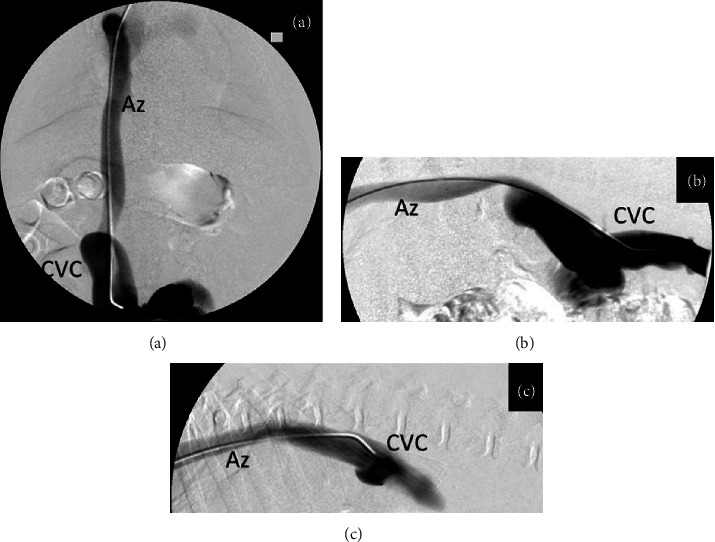
Digital subtraction angiography (DSA) images from selective venography for stent placement under fluoroscopic guidance: (a, b) ventrodorsal and laterolateral views and (c) right lateral view, after stent placement. Note the normalization of the cava shape.

The ventral aspect of the neck was aseptically prepared. A 7F introducer was percutaneously inserted in the right external jugular vein. A selective cavogram was performed through a multipurpose 0.035-inch catheter, with manual injection of 10 ml of iohexol 370 mgI/ml diluted with the same quantity of saline solution. The venography allowed identifying the vascular stenosis at the diaphragmatic level (Figure 3). The same jugular approach was used to deploy a self-expandable, nitinol, venous stent of 20 mm × 80 mm into the anomalous vessel across the stenosis. The introducer was then removed, and a manual compression of the site was performed for 15 minutes. A compressive neck bandage was placed for the next 12 hours.

Twenty-four hours after stent placement, the PU/CU was normal. Two days after stent placement, the dog was discharged from the hospital with instructions for recheck examination for one week after. Blood and urine analyses showed normalization of urea and creatinine levels, increased urine osmolality, and confirmed normal PU/CU (Table 1).

One year after the stent placement, the patient was completely reevaluated. A follow-up CT scan, made with the dog awake, showed reduced caval and right renal vein diameter. The left renal vein still had aneurysmal appearance. The left gonadal vein was still distended but not perfused. Other collaterals were not detected. There were no signs of thrombosis (Figures 1(c), 1(d), 2(c) and 2(d)). At the time of writing this report, three years after the initial diagnosis, the dog is still in good condition with normal renal function.

## 3. Discussion

To our knowledge, this is the first description of a percutaneous stent placement in a dog with symptomatic azygos continuation of the caudal vena cava. With this developmental venous anomaly, the systemic blood flow beyond the renal CVC was through a dilated azygos vein system (right azygos and hemiazygos veins), which emptied into the cranial vena cava and eventually into the right atrium. It is likely that many cavo-azygos communications go unnoticed as the dogs are asymptomatic. A previous case report in veterinary literature described a dog with similar vascular pattern showing lethargy, exercise intolerance, ascites, and venous collaterals [6]. In the present case, as well as in that previously described case, clinical signs were secondary to the effects of venous return obstruction and thrombus formation, rather than by the vascular variant itself. Collateral venous pathways found in the present dog, as well as in the previously described case in the literature, were indirect signs of the increased blood flow resistance in the CVC [7].

The venous hypertension in patients with chronic obstruction of the CVC blood flow to the heart leads to a wide spectrum of signs, depending on the level of obstruction and the efficiency of the veno-venous collaterals. Endovascular stenting of the posthepatic CVC was reported in Budd-Chiari syndrome as palliative treatment in three dogs with malignant postsinusoidal venous outflow obstruction and in a kitten with web-like obstruction of the hepatic CVC [8, 9].

In the dog of this report, the cavo-azygos channel showed a marked dorsoventral stenosis at the diaphragmatic passage. Turbulent blood flow below this passage and blood stasis likely caused a slow dilation of the CVC prerenal segment, iliac, and renal veins, with venous thrombosis formation. Both the stenosis and stasis can lead to thrombus formation for local inflammation (activation of endothelium, recruitment of immune cells and platelets to the vessel wall) or tissue factor and other coagulation and fibrinolysis-related mechanism [10].

The endovascular stenting of the cavo-azygos connection resulted in the rapid and dramatic improvement of the patient condition with relief of chronic CVC obstructive syndrome. Follow-up indicated a return of adequate renal perfusion and an absence of clinically significant permanent renal impairment. The percutaneous stenting of the stenotic segment of the cavo-azygos was safe, minimally invasive, and clinically effective.

## Figures and Tables

**Table 1:** The table resumes the BUN (blood urea nitrogen), the urine protein-to-creatinine ratio (UPCR), and the osmolality at the admission, the day after interventional radiology (IR) procedure, and 10 days after the stent placement.

Test	Admission	1 day after IR	10 days after IR	Min	Max	MU
BUN	70	18	39	19	53	mg/dl
Creatinine	2.08	1.37	1.29	0.75	1.33	mg/dl
UPCR	3.2	0.1	0.1	0.1	0.1	mg/dl
Osmolality	490	361	736	925	1934	mOsm/kg

MU: measurement unit.
